# Mapping the Risk of Anaemia in Preschool-Age Children: The Contribution of Malnutrition, Malaria, and Helminth Infections in West Africa

**DOI:** 10.1371/journal.pmed.1000438

**Published:** 2011-06-07

**Authors:** Ricardo J. Soares Magalhães, Archie C. A. Clements

**Affiliations:** School of Population Health, University of Queensland, Herston, Queensland, Australia; Kenya Medical Research Institute, Kenya

## Abstract

**Background:**

Childhood anaemia is considered a severe public health problem in most countries of sub-Saharan Africa. We investigated the geographical distribution of prevalence of anaemia and mean haemoglobin concentration (Hb) in children aged 1–4 y (preschool children) in West Africa. The aim was to estimate the geographical risk profile of anaemia accounting for malnutrition, malaria, and helminth infections, the risk of anaemia attributable to these factors, and the number of anaemia cases in preschool children for 2011.

**Methods and Findings:**

National cross-sectional household-based demographic health surveys were conducted in 7,147 children aged 1–4 y in Burkina Faso, Ghana, and Mali in 2003–2006. Bayesian geostatistical models were developed to predict the geographical distribution of mean Hb and anaemia risk, adjusting for the nutritional status of preschool children, the location of their residence, predicted *Plasmodium falciparum* parasite rate in the 2- to 10-y age group (*Pf* PR_2–10_), and predicted prevalence of *Schistosoma haematobium* and hookworm infections. In the four countries, prevalence of mild, moderate, and severe anaemia was 21%, 66%, and 13% in Burkina Faso; 28%, 65%, and 7% in Ghana, and 26%, 62%, and 12% in Mali. The mean Hb was lowest in Burkina Faso (89 g/l), in males (93 g/l), and for children 1–2 y (88 g/l). In West Africa, severe malnutrition, *Pf* PR_2–10_, and biological synergisms between *S. haematobium* and hookworm infections were significantly associated with anaemia risk; an estimated 36.8%, 14.9%, 3.7%, 4.2%, and 0.9% of anaemia cases could be averted by treating malnutrition, malaria, *S. haematobium* infections, hookworm infections, and *S. haematobium*/hookworm coinfections, respectively. A large spatial cluster of low mean Hb (<80 g/l) and maximal risk of anaemia (>95%) was predicted for an area shared by Burkina Faso and Mali. We estimate that in 2011, approximately 6.7 million children aged 1–4 y are anaemic in the three study countries.

**Conclusions:**

By mapping the distribution of anaemia risk in preschool children adjusted for malnutrition and parasitic infections, we provide a means to identify the geographical limits of anaemia burden and the contribution that malnutrition and parasites make to anaemia. Spatial targeting of ancillary micronutrient supplementation and control of other anaemia causes, such as malaria and helminth infection, can contribute to efficiently reducing the burden of anaemia in preschool children in Africa.

*Please see later in the article for the Editors' Summary*

## Abstract 2

Ricardo Soares Magalhães and colleagues used national cross-sectional household-based demographic health surveys to map the distribution of anemia risk in preschool children in Burkina Faso, Ghana, and Mali.

## Editors' Summary

**Background:**

Global estimates for the time period 1993–2005 suggest that that worldwide, nearly 300 million children had anemia, that is, hemoglobin levels less than 110 g/l. In sub-Saharan Africa, two-thirds of all children were anemic, representing 83.5 million children. These statistics are important because anemia in infancy and childhood is associated with poor cognitive development, reduced growth, problems with immune function—and ultimately, decreased survival. Malnutrition (including micronutrient deficiency, especially of iron, vitamin A, vitamin C, and folate), undernutrition, and infectious diseases, particularly HIV, malaria, and helminth infections (caused by hookworm and *Schistosoma haematobium*—which causes urinary schistosomiasis), are major causes of anemia in children. Although iron supplementation can often correct anemia, in some circumstances, iron deficiency can protect against common infectious agents, and giving iron can, on occasion, increase the severity of infectious disease in some children. Focusing on the treatment and prevention of infectious diseases that cause anemia is therefore an important alternative strategy in the treatment of anemia.

**Why Was This Study Done?:**

Control tools for targeting interventions for malaria and helminth infection in sub-Saharan Africa include modern spatial risk prediction methods that combine statistical models with geographical information systems (similar to those used in car navigation systems). However, to date no studies have used these tools to spatially predict the risk of anemia. Furthermore, the contribution that malnutrition and infections make to the overall anemia burden in Africa is largely unknown. In this study the researchers used these tools to predict the prevalence of anemia in three West African countries and to estimate the attributable risk of anemia due to malnutrition, malaria, and helminth infections.

**What Did the Researchers Do and Find?:**

The researchers used geographically linked data from the most recent Demographic and Health Surveys (DHS) in Burkina Faso (2003), Ghana (2003), and Mali (2006), which included capillary blood sampling and testing and detailed anthropometric (height and weight) measurements. A total of 7,147 children aged 1–4 years (3,477 girls and 3,670 boys) in the three countries were included in the analysis. The researchers mapped DHS survey locations in the three study countries using DHS cluster coordinates in a geographic information system. Using data from the Malaria Atlas Project, the researchers extracted spatially predicted values of *Plasmodium falciparum* parasite rate for each DHS cluster using a geographical information system and used previously reported parasitological survey data of hookworm and *S. haematobium* infections to predict helminth infection risk across the region. Then the researchers developed spatial prediction models using Bayesian statistics to estimate of the population attributable fraction for specific predictors for anemia. Data from the DHS showed that the prevalence of mild, moderate, and severe anemia was 21%, 66%, and 13% in Burkina Faso; 28%, 65%, and 7% in Ghana, and 26%, 62%, and 12% in Mali. The prevalence of stunting, wasting, and being underweight in the study area was 87.8%, 89.7%, and 71.2%, respectively, and the mean *P. falciparum* parasite rate, and rates of *S. haematobium* infection, hookworm infection, and *S. haematobium*/hookworm coinfection for the study area were 52.0%, 26.8%, 8.2%, and 3.6%, respectively. The overall results indicate that in the three countries, approximately 6.7 million children aged 1–4 years have anemia. Severe malnutrition, *P. falciparum* infection, hookworm infection, *S. haematobium* infection, and hookworm/*S. haematobium* coinfection were responsible for an estimated 2.5 million, 1.0 million, 250,000, 285,000, and 61,000 anemia cases, respectively. Central Burkina Faso and southern Ghana had the highest number of anemic children.

**What Do These Findings Mean?:**

These results add insight and detail to anemia prevalence and anemia severity within different geographical areas in three West African countries. The combination of anemia and mean hemoglobin predictive maps identifies communities in West Africa where preschool-age children are at increased risk of morbidity. The use of anemia maps has important practical implications for targeted control in these countries, such as guiding the efficient allocation of nutrient supplements and fortified foods, and enabling risk assessment of anemia due to different causes, which would in turn constitute an evidence base to calculate the best balance between interventions.

**Additional Information:**

Please access these Web sites via the online version of this summary at http://dx.doi.org/10.1371/journal.pmed.1000438.

- This study is further discussed in a *PLoS Medicine* Perspective by Abdisalan Noor
- The WHO Web site has comprehensive information on the worldwide prevalence of anemia
- More information on Demographic Health Surveys is available
- More information on global predictions of malaria is available

## Introduction

The most up-to-date global estimates of childhood anaemia indicate that 293.1 million children aged <5 y are anaemic worldwide, and 28.5% of those are located in sub-Saharan Africa (SSA) [1]. Childhood anaemia is considered a severe public health problem in SSA, reaching 67% prevalence, or 83.5 million children, in the region [1]. Anaemia in infancy and childhood is associated with reduced cognitive development [2], growth [3], immune function [4], and survival.

Anaemia is usually multifactorial in origin, and malnutrition, infectious diseases, inherited haemoglobinopathies [5], and thalassemias [6] are thought to be the major contributors.

The main micronutrient deficiency contributing to anaemia is iron deficiency [7], but other micronutrients, such as vitamin A [8], vitamin C [9], and folate [10] are important in the pathophysiology of anaemia. Among the most common infectious diseases in SSA, malaria [11], HIV [12], bacteraemia caused by organisms such as *Steptococcus pneumoniae*, non-typhi *Salmonella* species, and *Haemophilus influenzae* type b [13],[14], and helminth infections caused by hookworm and *Schistosoma haematobium* (the aetiological agent of urinary schistosomiasis) [15]–[17] are known to cause anaemia. The general mechanisms by which these infections lead to anaemia include blood loss, sequestration of red blood cells by the spleen, haemolysis by antibodies, and anaemia of inflammation (via TNF-alpha and IL-6 production) [18],[19]. In the case of parasite infections, synergisms between multiple species infections (coinfection) and high parasite burden (infection intensity) are known to exacerbate anaemia [20]–[22].

The most common form of anaemia is caused by low levels of iron (or iron-deficiency anaemia), and efforts to mitigate its effects include the population delivery of iron supplements and food fortification with iron [23]. However, in addition to undernutrition, immune responses to infections can lead to infection-induced hypoferremia, which prevents the growth of pathogens and can be anti-inflammatory by reducing a potential prooxidant. This well-recognised phenomenon shows that iron deficiency can protect against common infectious agents, and recent empirical evidence suggests iron supplementation is linked to increased severity of infectious disease in the presence of malaria and/or undernutrition in certain subgroups [24]. Anaemia cases in which blood haemoglobin concentration (Hb) falls below 70 g/l are potentially life-threatening situations, and control can be achieved by providing hospital emergency treatment, which includes iron and folate supplementation and blood transfusions [25].

Anaemia control can also focus on infectious disease causes of anaemia. In the case of malaria control programmes, the adoption of artemisinin-based combination therapies for the treatment of malaria patients and the large-scale deployment of long-lasting insecticide-treated bed nets among high-risk groups, especially young children and pregnant women, are currently being promoted [26],[27]. An alternative strategy is chemoprophylaxis with antimalarial drugs: intermittent preventive treatment for women in pregnancy [28] and once-a-term mass administration of a full therapeutic course of antimalarial drugs to schoolchildren [29] are effective at reducing malaria parasitaemia and halving the rates of anaemia among these high-risk groups. The fundamental aim of the helminth control programmes is morbidity control, and the prevalence of anaemia has been used as a measurable target in control programmes for schistosomiasis and soil-transmitted helminths [30]. The basis of control of helminth infections is mass administration of single-dose antihelminthics [31]. In areas with high prevalence of helminth infection, treatment of severe anaemia cases generally includes deworming [32]. However, supplementation has been found to be inefficient in the presence of inflammation due to iron sequestration, and deworming is warranted when anaemia coexists with high parasite prevalence [25]. Mass deworming has been shown to cause a small increase in Hb in preschool and school-age children in SSA [33]. With the aim of alleviating the anaemia burden of endemic populations, micronutrients are also being distributed as part of deworming programmes [34]. For example, vitamin A supplementation is being given to preschool and school-age children in many countries in Africa as a single dose immediately after deworming [35].

Targeting the correct set of interventions to population subgroups at increased risk of anaemia would have important implications in more efficient delivery of limited national resources. Modern spatial risk prediction methods are being used as control tools for targeting malaria [36] and helminth infection [37] interventions in SSA. To date there are no studies that have predicted spatially the risk of anaemia. Furthermore, the contribution that malnutrition and infections make to the overall anaemia burden is largely unknown. Population attributable fractions (PAFs) are useful for translating surveillance data on risk factor prevalence and disease occurrence into numbers that can help policymakers and the public appreciate the potential benefits to be gained by risk factor reduction and health promotion [38]. This information could provide an important evidence base to work out the best delivery balance between micronutrient supplementation and food fortification versus deworming and malaria control.

In this paper, we describe unique preschool anaemia data from national surveys in three contiguous countries in the West African region (Burkina Faso, Ghana, and Mali) and predict, to our knowledge for the first time, the prevalence of anaemia and mean Hb across the region. In doing so, we adjust for malnutrition and the prevalence of infection of the major parasitic contributors and estimate the attributable risk of anaemia due to malnutrition, malaria, and helminth infections. We aimed to develop a predictive decision-support tool for quantifying the overall burden of anaemia, spatial heterogeneity in the anaemia burden, and the contribution that malnutrition and parasitic infections make to preschool anaemia.

## Methods

### Data

The preschool anaemia data used in this study were collected within the Demographic and Health Surveys (DHS) programme. These datasets are in the public domain and are available from Measure DHS (http://www.measuredhs.com/login.cfm) on demand. Anaemia data were collected by the MEASURE DHS+ programme using standardised protocols and anaemia testing procedures [39]. Capillary blood samples in young children were obtained by heel prick and were tested using the Hemocue blood haemoglobin testing system, which is considered a durable and reliable system under field conditions [39]. More detailed information on DHS survey design and anaemia testing data are available online (http://www.measuredhs.com) and is summarised in Text S1.

Anthropometric measures (height-for-age *Z*-score, an indicator of stunting; weight-for-height *Z*-score, an indicator of wasting; and weight-for-age *Z*-score, an indicator of underweight) and data on anaemia status for children aged 1–4 y were extracted from the DHS household survey datasets for Burkina Faso (2003), Ghana (2003), and Mali (2006). Although these surveys included data for children aged <1 y, we selected children aged 1–4 y only since children aged <1 y are known to experience a physiological decrease of Hb [11] and Hb in children 0–1 is dependent on maternal iron provisioning and therefore is likely to be confounded by maternal anaemia status—these physiologic factors would inhibit accurate estimation of anaemia risk in infants. To classify the undernutrition of preschool-age children we used composite index of anthropometric failure (CIAF) groupings, which provide a summary statistic of anthropometric failures [40]. The CIAF is a method of partitioning undernutrition in children into seven mutually exclusive categories including no anthropometric failure (CIAF Group A), single anthropometric failures (stunting only, CIAF Group F; wasting only, CIAF Group B; and underweight only, CIAF Group Y), and multiple anthropometric failures (stunting and underweight, CIAF Group E; wasting and underweight, CIAF Group C; and wasting, stunting, and underweight, CIAF Group D).

The geographical unit of the DHS surveys is the sample “cluster”. These are usually census enumeration areas, sometimes villages in rural areas or city blocks in urban areas. Coordinates taken at the centre of each cluster were used to geo-locate clusters in the three study countries. We extracted spatially predicted values of *P. falciparum* parasite rate in the 2- to 10-y age group (*Pf* PR_2–10_) for each DHS cluster using the geographical information system ArcView version 9.3 (ESRI). These spatial predictions were created by the Malaria Atlas Project (http://www.map.ox.ac.uk/) using model-based geostatistics (MBG); the *Pf* PR_2–10_ was estimated based on data from microscopy (approximately 80%) and rapid diagnostic tests (approximately 20%) [36]. We used previously reported parasitological survey data of hookworm and *S. haematobium* infections in Burkina Faso, Ghana, and Mali and MBG [37],[41],[42] to predict helminth infection risk across the region. Data for preschool-age children were not collected in these parasitic surveys, and predictions specific to the 1- to 4-y age group were, therefore, not available. We age-standardised the spatial prediction maps available for the 5- to 9-y age group to the 1- to 4-y age group based on age-prevalence profiles of these infections (more detail in Text S1). Spatially predicted values of prevalence of infection and coinfection with *S. haematobium* and hookworm in children aged 1–4 y were then extracted for each DHS cluster in the geographical information system for spatial modelling. A 5×5 km resolution rural/urban surface derived from the Global Rural-Urban Mapping Project beta product was obtained from the Center for International Earth Science Information Network of the Earth Institute at Columbia University (http://sedac.ciesin.columbia.edu/gpw/global.jsp). The values of this surface were extracted for each DHS survey cluster in the geographical information system to define whether the residence was urban or rural.

### Spatial Risk Prediction

Blood Hb is the key indicator for anaemia, and different age groups have different cut-off points for the haemoglobin level below which an individual is classified as anaemic [23]. A cut-off of <110 g/l was used to define anaemia in children aged 1–4 y, based on altitude-adjusted Hb available in a continuous scale. Within the group of anaemic individuals, the severity level can also be defined by clinically relevant altitude-adjusted Hb cut-offs: mild anaemia, 100–109 g/l; moderate anaemia, 70–99 g/l; and severe anaemia, < 70 g/l [23]. The initial candidate set of predictor variables included gender, age in months, number of members in the household, residence (rural/urban), CIAF group, and the cluster-level ecological variables of *Pf* PR_2–10_, prevalence of *S. haematobium* infection, prevalence of hookworm infection, and prevalence of *S. haematobium* and hookworm mono- and coinfection.

We developed spatial prediction models using the Bayesian statistical software WinBUGS version 1.4 (Medical Research Council Biostatistics Unit and Imperial College London). All models had the individual covariates plus a geostatistical random effect, in which spatial autocorrelation between locations was modelled using an exponentially decaying autocorrelation function (Text S1). Model selection for the prediction stage was based on the evaluation of the deviance information criteria (DIC) of each model (the lower the DIC, the better the model fit). Spatial prediction was based on MBG, using the model with the lowest DIC [43]. Statistical notation of Bayesian geostatistical models and spatial interpolation procedures are presented in Text S1.

### Model Validation

To assess the predictive performance of the final models of prevalence of anaemia and Hb, a single validation dataset was generated by random selection of 25% of the data (more detail in Text S1). The ability of the models to predict known mean prevalence of anaemia and mean Hb was assessed by three summary statistics: mean prediction error, mean absolute prediction error, and the correlation coefficient between the predicted and the actual values. The mean prediction error provides a measure of the bias of the predictor, the mean absolute prediction error provides a measure of the mean accuracy of individual predictions, and the correlation coefficient provides a measure of association between the observed data and prediction sets. The correlation between the observed and prediction data were visualised using scatter plots with a least-squares best fitting line and 95% confidence intervals. The ability of the final model to predict anaemia endemicity class membership was assessed by comparing the predicted prevalence of anaemia to the observed prevalence, dichotomised at 80%. Following the same procedure, the predicted mean Hb was compared to the observed mean Hb, dichotomised at 90 g/l. The area under curve (AUC) statistic of the receiver operating characteristic curve was used for the comparison [44]. An AUC value of 0.7 was taken to indicate acceptable predictive performance.

### Estimation of the Number of Children Aged under 5 y with Anaemia and the Population Attributable Fraction of Anaemia Due to Different Contributors

We extracted population density data (total heads per 2.5 arc-minute grid cell) for Burkina Faso, Ghana, and Mali from the Gridded Population of the World (GPWv3) map for 2009 [45]. The population structure and population growth rate of each country was obtained from the World Population Prospects 2008 Revision Population Database (Population Division of the Department of Economic and Social Affairs of the United Nations Secretariat; http://esa.un.org/unpd/wpp/unpp/panel_population.htm). However, the age categories were slightly different to those used in our analysis. The proportion aged 0–4 y obtained from the World Population Prospects database was discounted by a factor of 0.2 to obtain the proportion aged 1–4 y (the age group in our study). The population density map was multiplied by the proportion of the population aged 1–4 y in each country and by the estimated population growth rate for the period 2005–2011 to derive a map of the number of children aged 1–4 y in 2011 in each grid cell.

Estimates of the PAF for specific predictors are used to guide policymakers in planning public health interventions [46]. Estimation procedures for PAF of anaemia for helminth infections in the 1- to 4-y age group are presented in Text S1.

## Results

### Survey Results

**Figure 1:**
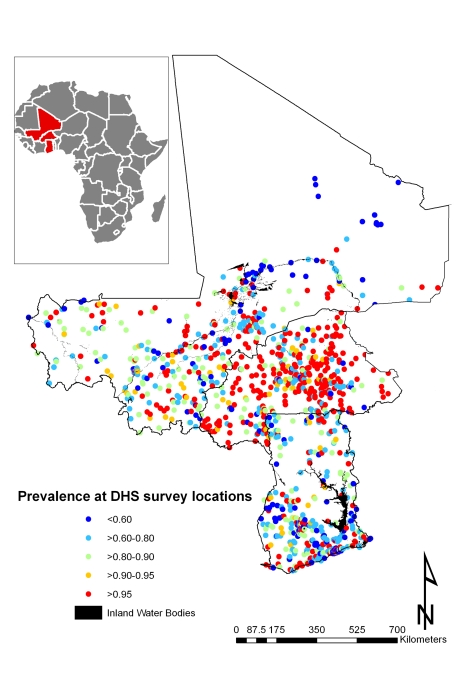
Mean prevalence of anaemia at 1,192 DHS survey sites. Surveys conducted in Burkina Faso (2003), Ghana (2003), and Mali (2006).

**Figure 2:**
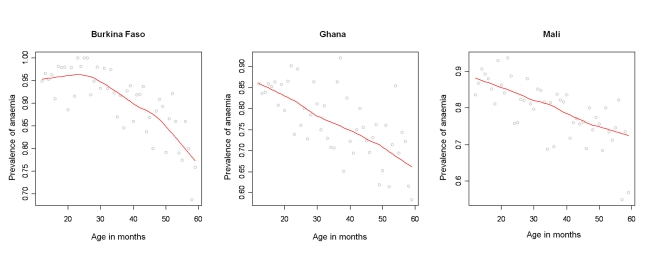
Profile of anaemia by age in Burkina Faso, Ghana, and Mali. Anaemia (*y*-axis; Hb<110 g/l) by age in months (*x*-axis; in months) with smooth fit line (red line) generated by a loess smoother, in children aged 1–4 y in the DHS surveys for Burkina Faso (2003), Ghana (2003), and Mali (2006).

**Figure 3:**
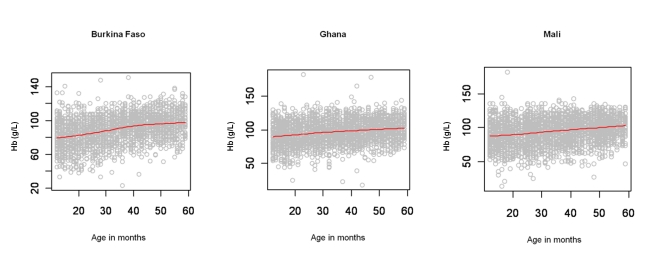
Age patterns of mean haemoglobin concentration. Age (*x*-axis; in months) patterns of mean Hb (*y*-axis; g/l) with smooth fit line (red line) generated by a loess smoother, in children aged 1–4 y in the DHS surveys for Burkina Faso (2003), Ghana (2003), and Mali (2006).

A total of 7,147 children aged 1–4 y, including 3,477 girls and 3,670 boys, in Burkina Faso (2,096 children), Ghana (2,360 children), and Mali (2,691 children) were included in the analysis. We included in the analysis all children with complete geographical (i.e., DHS cluster coordinates), demographical (i.e., age, gender, and number of members in household), and morbidity (i.e., Hb and malnutrition status) information. The mean age in months was 34.4 (standard deviation [SD]: 13.7), and the mean number of members per household was 7.7 (SD: 4.4). The spatial distribution of the raw prevalence of anaemia at 1,192 locations in the study area is presented in Figure 1. Results from the DHS data used show that the prevalence of mild, moderate, and severe anaemia was 21%, 66%, and 13% in Burkina Faso; 28%, 65%, and 7% in Ghana, and 26%, 62%, and 12% in Mali (Table 1). There was a significant difference in the proportion of mild anaemia between Burkina Faso and the other countries (*p = *0.015), and there was also a significant difference in the proportion of severe anaemia between Burkina Faso and Ghana (*p = *0.021), but not between Burkina Faso and Mali. None of the remainding geographical differences in anaemia levels were significant. The results indicate that prevalence of anaemia is highest in children aged 1–2 y and decreases with increasing age (Figure 2). By contrast, Hb steadily increases with age (Figure 3). The mean Hb was lower in Burkina Faso (89 g/l) than in Ghana (97 g/l) (*p = *0.027) and Mali (94 g/l) (*p = *0.047). It was lower in males (94 g/l) than females (96 g/l) (*p>*0.05), and for children aged 1–2 y (87 g/l) than for children aged 2+ y (99 g/l) (*p<*0.001). The prevalence of stunting, wasting, and being underweight in the study area was 87.8%, 89.7%, and 71.2%, respectively. The prevalence of anthropometric failures based on CIAF groupings was the following: 3.3% for no anthropometric failures (Group A), 7.1% for single failures (Groups Y and F), 7.0% for wasting and underweight (Group C), and 62.4% for wasting and stunting and underweight (Group D). The mean *Pf* PR_2–10_ and rates of *S. haematobium* infection, hookworm infection, and *S. haematobium*/hookworm coinfection for the study area were 52.0% (SD: 12.5), 26.8% (SD: 19.1), 8.2% (SD: 10.0), and 3.6% (SD: 5.7), respectively.

**Table 1:** Number and proportion of children aged 1–4 y with mild anaemia, moderate anaemia, and severe anaemia in 5,888 anaemic children in the West Africa region.

Background Characteristic	Mild Anaemia			Moderate Anaemia			Severe Anaemia		
	Burkina Faso	Ghana	Mali	Burkina Faso	Ghana	Mali	Burkina Faso	Ghana	Mali
**Age**									
1–2 y	50 (0.12)	118 (0.23)	109 (0.20)	327 (0.26)	361 (0.30)	406 (0.30)	137 (0.57)	66 (0.55)	111 (0.43)
2+ y	353 (0.88)	404 (0.77)	448 (0.80)	941 (0.74)	825 (0.70)	930 (0.70)	103 (0.43)	54 (0.45)	145 (0.57)
**Sex**									
Male	205 (0.51)	251 (0.48)	297 (0.53)	616 (0.49)	579 (0.49)	628 (0.47)	109 (0.45)	61 (0.51)	114 (0.44)
Female	198 (0.49)	271 (0.52)	260 (0.47)	652 (0.51)	607 (0.51)	708 (0.53)	131 (0.55)	59 (0.49)	142 (0.56)
**Household size**									
2–7 members	132 (0.33)	339 (0.65)	280 (0.50)	407 (0.32)	731 (0.62)	603 (0.45)	80 (0.33)	60 (0.50)	111 (0.43)
7+ members	271 (0.67)	183 (0.35)	277 (0.50)	861 (0.68)	455 (0.38)	733 (0.55)	160 (0.67)	60 (0.50)	145 (0.57)
**Overall** a	403 (0.21)	522 (0.29)	557 (0.26)	1,268 (0.66)	1,186 (0.65)	1,336 (0.62)	240 (0.13)	120 (0.07)	256 (0.12)

Mild anaemia defined as 100–109 g/l; moderate anaemia, as 70–99 g/l, and severe anaemia, as <70 g/l. West Africa region includes Burkina Faso (*n = *1,911), Ghana (*n = *1,828), and Mali (*n = *2,149).

a Numbers in parentheses are the proportion of anaemia cases in the country.

### Predicted Risk of Childhood Anaemia

**Figure 4:**
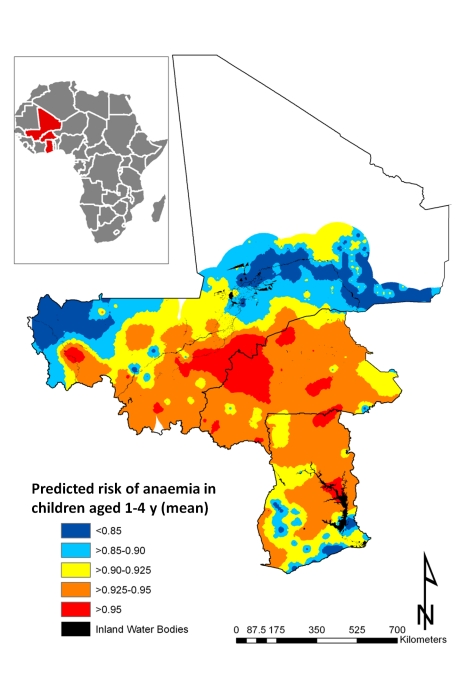
Predictive geographical risk of anaemia in children aged 1–4 y, based on a model-based geostatistical Bernoulli model.

It can be seen from the 95% credible interval, that individual-level variables significantly associated with risk of anaemia in all models tested are age in months, residence (rural versus urban), and having three anthropometric failures (CIAF Group D); gender, the number of members in the household, and other CIAF groupings were not associated with anaemia risk (Table 2). In all models tested, the *Pf* PR_2–10_ was significantly and positively associated with anaemia risk. In model 6, the fixed effect of prevalence of hookworm and the product term between the prevalence of *S. haematobium* and the prevalence of hookworm infection were significantly and positively associated with risk of anaemia. While not significant at the 5% level, prevalence of *S. haematobium* (models 4 and 6) and the prevalence of coinfection (model 3) were positively associated with anaemia. The model with the lowest DIC was model 6, and, therefore, this model was used in the prediction phase. This model was able to predict prevalence of anaemia being greater that 80% with an AUC>0.8 (Table 3). The risk of anaemia in children aged 1–4 y was consistently high across the entire study area, with maximal prevalence (>95%) in a large focus straddling the borders of Burkina Faso and Mali (Figure 4). Smaller sized foci of high prevalence of anaemia were also predicted for southern areas of Mali, central areas of Burkina Faso, northern areas in Ghana, and areas adjacent to Volta Lake in Ghana. Phi (ϕ) indicates the rate of decay of spatial autocorrelation and varied from 13.68 in model 3 to 14.80 in model 5. Therefore, after accounting for the effect of covariates in model 6, the radii of the foci were approximately 23 km (note, ϕ is measured in decimal degrees and 3/ϕ determines the cluster size; one decimal degree is approximately 111 km at the equator).

**Table 2:** Associations with anaemia risk, based on model-based geostatistical Bernoulli models.

Variable	Model 1	Model 2	Model 3	Model 4	Model 5	Model 6
Male (versus Female)	0.09 (−0.15, 0.31)	0.09 (−0.15, 0.32)	0.09 (−0.15, 0.32)	0.09 (−0.14, 0.32)	0.10 (−0.14, 0.32)	0.09 (−0.14, 0.231)
Number of members in household^a^	−0.04 (−0.08, 0.01)	−0.04 (−0.08, 0.007)	−0.04 (−0.08, 0.006)	−0.04 (−0.08, 0.01)	−0.04 (−0.08, 0.007)	−0.04 (−0.08, 0.007)
Age in months^a^	−0.03 (−0.05, −0.01)	−0.03 (−0.05, −0.01)	−0.03 (−0.05, −0.01)	−0.03 (−0.05, −0.01)	−0.03(−0.05, −0.01)	−0.03 (−0.05, −0.01)
Rural (versus urban)	0.69 (0.27, 1.13)	0.68 (0.26, 1.11)	0.69 (0.24, 1.13)	0.67 (0.24, 1.11)	0.67 (0.25, 1.10)	0.69 (0.27, 1.11)
CIAF Groups Y and F (versus CIAF Group A)	−0.19 (−0.79, 0.43)	−0.19 (−0.76, 0.43)	−0.17 (−0.76, 0.45)	−0.20 (−0.78, 0.41)	−0.19 (−0.75, 0.38)	−0.02 (−0.77, 0.41)
CIAF Group C (versus CIAF Group A)	0.11 (−0.44, 0.64)	0.10 (−0.41, 0.63)	0.13 (−0.42, 0.66)	0.10 (−0.44, 0.63)	0.11 (−0.41, 0.59)	0.10 (−0.42, 0.60)
CIAF Group D (versus CIAF Group A)	0.67 (0.13, 1.19)	0.66 (0.15, 1.19)	0.66 (0.12, 1.19)	0.66 (0.13, 1.18)	0.66 (0.16, 1.14)	0.66 (0.15, 1.16)
*Pf*PR_2–10_ a	0.37 (0.17, 0.57)	0.32 (0.11, 0.53)	0.30 (0.04, 0.51)	0.36 (0.17, 0.56)	0.28 (0.03, 0.49)	0.29 (0.04, 0.50)
Prevalence of *S. haematobium* monoinfections^a^	0.05 (−0.13, 0.25)					
Prevalence of hookworm monoinfections^a^		0.11 (−0.09, 0.33)				
*S. haematobium/*hookworm coinfection^a^			0.23 (−0.05, 0.53)			
Prevalence of *S. haematobium* a				0.05 (−0.13, 0.25)		0.11 (−0.09, 0.32)
Prevalence of hookworm^a^					0.22 (0.03, 0.47)	0.35 (0.11, 0.67)
Interaction: prevalence of *S. haematobium ×* prevalence of hookworm^a^						0.28 (0.04, 0.57)
Intercept	1.61 (0.95, 2.37)	1.60 (0.94, 2.31)	1.59 (0.88, 2.25)	1.62 (0.91, 2.23)	1.64 (1.02, 2.28)	1.63 (0.97, 2.29)
ϕ (rate of decay of spatial correlation)	14.24 (3.14, 19.73)	13.80 (2.80, 19.73)	13.68 (1.86, 19.68)	15.38 (3.23, 19.71)	14.80 (2.80, 19.71)	14.25 (2.01, 19.63)
σ^2^ (variance of spatial random effect)	1.45 (0.97, 1.95)	1.45 (0.95, 1.97)	1.36 (0.91, 1.98)	1.32 (0.01, 1.94)	1.40 (0.91, 1.98)	1.39 (0.92, 1.97)
DIC	2,584.4	2,583.5	2,587.8	2,586.5	2,583.7	2,572.5

Association values are given as posterior mean (95% Bayesian credible interval).

a Variables were standardised to have mean* = *0 and SD* = *1.

**Table 3:** Summary of validation statistics for predictive models of anaemia prevalence and haemoglobin concentration in Burkina Faso, Ghana, and Mali.

Validation Measure	Prevalence of Anaemia	Haemoglobin Concentration
Area under the ROC curve (95% CI)	0.82 (0.75, 0.88)	0.77 (0.69, 0.83)
Mean error^a^	0.03 (4.88)	−7.99 (9.36)
Mean absolute error^a^	0.12 (18.57)	10.96 (12.83)
Correlation	0.79	0.82

a The observed values were compared to the mean of the posterior distribution of the each predicted value of prevalence of anaemia and Hb. The estimates in parenthesis are the percentage of the overall mean attributed to the error estimate.

CI, confidence interval; ROC, receiver operating characteristic.

### Predicted Mean Haemoglobin Concentration

**Figure 5:**
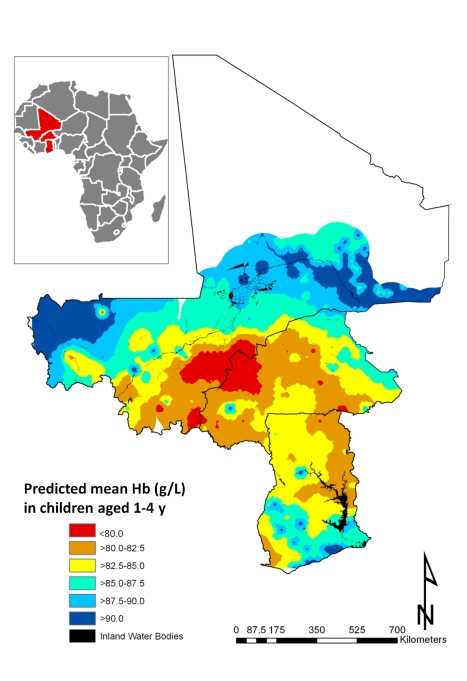
Predictive geographical variation of mean haemoglobin concentration in children aged 1–4 y, based on a model-based geostatistical Gaussian model.

All individual-level variables except number of members in household, age in months, and single anthropometric failures (CIAF Groups Y and F) were significantly associated with mean Hb in all models tested (Table 4). While rural residences and two or more anthropometric failures were significantly and negatively associated with the mean Hb, there was a significant positive association with mean Hb in male children. As with the models of risk of anaemia, *Pf* PR_2–10_ was significantly associated with mean Hb in all models tested. At the 5% level neither *S. haematobium* nor hookworm infection was significantly associated with mean Hb; however, *S. haematobium/*hookworm coinfections, hookworm monoinfections, and hookworm prevalence of infection were negatively associated with mean Hb. Estimates presented in Figure 5 are the mean posterior predicted mean Hb values from model 6 (the model that yielded the lowest DIC); this model was able to predict Hb greater than 90 g/l with an AUC >0.7 (Table 3). Figure 5 shows overlapping similarities to the map showing the predicted risk of anaemia (Figure 4) in that areas where Hb was predicted to be lowest (<80 g/l) for children 1–4 y are localised in a large focus straddling the borders of Burkina Faso and Mali. After accounting for the effect of covariates in model 6, the radii of the foci were approximately 22 km (Table 4).

**Table 4:** Associations with altitude-adjusted haemoglobin concentration, based on model-based geostatistical Gaussian models.

Variable	Model 1	Model 2	Model 3	Model 4	Model 5	Model 6
Male (versus Female)	0.13 (0.13, 0.23)	0.19 (0.15, 0.23)	0.19 (0.15, 0.24)	0.19 (0.15, 0.23)	0.19 (0.15, 0.23)	0.19 (0.15, 0.23)
Number of members in household^a^	−0.64 (−1.87, 0.67)	−0.64 (−1.86, 0.67)	−0.60 (−1.85, 0.69)	−0.61 (−1.71, 0.66)	−0.65 (−1.83, 0.67)	−0.69 (−1.79, 0.70)
Age in months^a^	0.12 (−0.10, 0.28)	0.13 (−0.06, 0.28)	0.13 (−0.05, 0.29)	0.13 (−0.04, 0.29)	0.11 (−0.07, 0.29)	0.15 (−0.01, 0.30)
Rural (versus urban)	−4.71 (−7.24, −2.14)	−4.70 (−7.55, −2.19)	−4.67 (−7.41, −2.03)	−4.62 (−7.39, −2.02)	−4.75 (−7.43, −2.24)	−4.69 (−7.39, −1.89)
CIAF Groups Y and F (versus CIAF Group A)	0.78 (−2.48, 4.29)	0.76 (−2.68, 4.28)	0.77 (−2.62, 4.25)	0.79 (−2.55, 4.31)	0.82 (−2.40, 4.42)	0.69 (−2.55, 4.09)
CIAF Group C (versus CIAF Group A)	−3.45 (−6.50, −0.47)	−3.51 (−6.61, −0.53)	−3.21 (−6.47, −0.27)	−3.52 (−6.57, −0.57)	−3.53 (−6.53, −0.47)	−3.59 (−6.64, −0.37)
CIAF Group D (versus CIAF Group A)	−7.85 (−10.35, −4.78)	−7.83 (−10.39, −4.72)	−7.81 (−10.37, −4.64)	−7.80 (−10.30, −4.61)	−7.79 (−10.43, −4.60)	−7.92 (−10.42, −4.58)
*Pf*PR_2–10_ a	−2.15 (−3.19, −1.17)	−1.81 (−2.93, −0.78)	−2.11 (−3.20, −1.07)	−2.15 (−3.15, −1.11)	−1.85 (−2.93, −0.83)	−1.82 (−3.08, −0.81)
Prevalence of *S. haematobium* monoinfections^a^	0.10 (−0.76, 1.23)					
Prevalence of hookworm monoinfections^a^		−0.87 (−1.94, 0.08)				
*S. haematobium/*hookworm coinfection^a^			−0.40 (−1.48, 0.52)			
Prevalence of *S. haematobium* a				0.17 (−0.82, 1.37)		0.15 (−0.76, 1.05)
Prevalence of hookworm^a^					−0.98 (−1.89, 0.01)	−1.09 (−2.23, −0.04)
Interaction: prevalence of *S. haematobium ×* prevalence of hookworm^a^						−0.37 (−1.31, 0.64)
Intercept	99.85 (94.74, 102.5)	99.81 (94.83, 102.3)	97.62 (94.78, 102.5)	98.41 (94.82, 102.6)	98.54 (94.12, 102.7)	98.97 (94.85, 102.3)
ϕ (rate of decay of spatial correlation)	15.43 (7.59, 18.22)	15.23 (8.59, 17.92)	15.75 (8.12, 18.21)	15.51 (8.07, 18.29)	14.78 (8.38, 18.57)	15.24 (9.53, 18.68)
σ^2^ (variance of spatial random effect)	45.51 (36.78, 68.14)	46.91 (36.89, 68.08)	47.55 (35.75, 67.97)	47.91 (34.56, 68.67)	40.98 (26.22, 66.97)	54.64 (37.42, 69.98)
DIC	26,544.5	26,543.1	26,545.4	26,545.8	26,671.7	26,533.7

Association values are given as posterior mean (95% Bayesian credible interval).

a Variables were standardised to have mean* = *0 and SD* = *1.

### Risk of anaemia attributable to *S. haematobium* and hookworm infections

We estimated the PAF of anaemia due to CIAF Group D, *P. falciparum*, *S. haematobium,* and hookworm separately from model 6, and that due to coinfections from Model 3. Our results indicate that the estimated risk of anaemia attributable to CIAF Group D, *P. falciparum*, *S. haematobium*, hookworm, and *S. haematobium*/hookworm coinfection is 36.8%, 14.9%, 3.7%, 4.2%, and 0.9%, respectively.

### Number of children and geographical distribution of childhood anaemia

**Figure 6:**
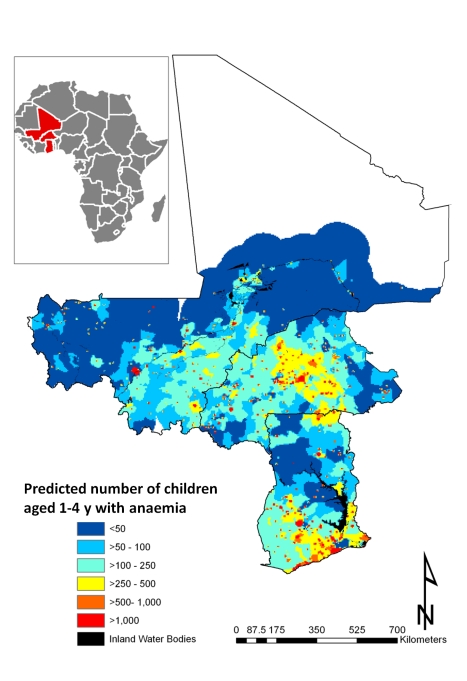
Predictive geographical variation of number of children aged 1–4 y with anaemia, for 2011.

The predicted total number of children aged 1–4 y with anaemia in Burkina Faso, Ghana, and Mali for 2011 is presented in Table 5. Our results indicate that in the three countries, approximately 6.7 million children aged 1–4 y are anaemic. Severe malnutrition, *P. falciparum* infection, hookworm infection, *S. haematobium* infection, and *S. haematobium*/hookworm coinfection were responsible for an estimated 2.5 million, 1.0 million, 250,000, 285,000, and 61,000 anaemia cases, respectively, in 2011. The areas with the greatest predicted number of anaemic children are located central Burkina Faso and southern Ghana (Figure 6). Figure 6 shows the number of children with anaemia in each 5×5 km pixel.

**Table 5:** Predicted number of children aged 1–4 y with anaemia in Burkina Faso, Ghana, and Mali in 2011.

Country	Total Population for 2009 (in Thousands)^a^	Annual Population Growth Rate for 2005–2011 (Percent)^b^	Percentage of Children Aged 1–4 y^c^	Number of Children Aged 1–4 y with Anaemia (in Thousands)	Percentage of Children Aged 1–4 y with Anaemia
Burkina Faso	15,922	3.39	15.7	2,585	90.51
Ghana	22,547	2.09	11.2	2,400	87.50
Mali	13,588	2.37	13.8	1,792	87.03

a Source: based on a 2.5 arc-minute resolution Gridded Population of the World (GPWv3) map for 2009.

b Source: World Population Prospects 2008 Revision Population Database (Population Division of the Department of Economic and Social Affairs of the United Nations Secretariat; http://esa.un.org/unpp).

c The estimates provided by the World Population Prospects database are for children aged 0–4.99 y. To obtain estimates of the percentage of children aged 1–4 y (the age group included in our analysis), the estimates presented by the World Population Prospects database were discounted by a factor of 0.2.

## Discussion

This study presents new cartographic resources that shed new light on the ranking of anaemia prevalence and anaemia severity within the countries studied by depicting important sub-national geographical heterogeneities, representing an added value over and above what could be achieved directly from national-level summary statistics of the DHS data alone. The approach addresses important operational constraints for anaemia control in the African continent, and the resulting maps could provide the next step needed for efficient and effective anaemia control in preschool children in the following ways. First, they could be used by national programme managers as decision-support tools for targeting the delivery of ancillary micronutrient supplementation and fortified food, with the aim of reducing iron-deficiency anaemia. Second, empirical maps of anaemia in this age group would allow the identification of subgroups where the secondary effects of micronutrient supplementation could be minimised. For example, the main concern about iron supplementation is the fact that it has been linked to increased severity of infectious disease in the presence of malaria and/or undernutrition in preschool children [24]. Finally, anaemia maps would allow the monitoring and evaluation of the impact of anaemia control programmes and, in the case of severe anaemia, planning resource allocation to combat life-threatening anaemia [37].

### Burden of Childhood Anaemia for West Africa in 2011

Based on the World Health Organization classification system for anaemia prevalence [23], it is clear that anaemia is a severe public health problem in the study area. We demonstrated that anaemia risk in children aged 1–4 y is high throughout the study area, with the highest risk in a large region extending across the borders of Burkina Faso and Mali. We predicted that the number of childhood anaemia cases is highest in Burkina Faso, followed by Ghana and Mali, and the magnitude of our predictions is consistent with estimates recently reported by the World Health Organization [1]. Using a predictive map of mean Hb we have shown that areas of severe anaemia are much smaller but overlap with areas where the prevalence of anaemia was predicted to be highest (>95%). These results suggest that resources for the treatment of moderate to severe anaemia, such as iron supplementation, deworming, and blood for emergency transfusion, should be prioritised towards populations located in the clusters of high anaemia risk identified in this study.

This study reveals that malnutrition plays a central role in preschool anaemia burden in West Africa. The model including malnutrition, *Pf*PR_2–10_, and helminth coinfection (Model 6) indicates that almost 40% of anaemia cases in preschool children in 2011 would have been averted by improving the nutritional status of children. Socio-economic status is a well-known risk factor for anaemia and infection at small spatial scales [47], and our results show that rural households are at significantly increased risk of anaemia compared to urban households. The same model also underlines the role of malaria infection in preschool children anaemia burden in the West African region in that the proportion of anaemia attributable to malaria was approximately 15%. These results are supported by earlier findings in Kenya using individual-level data (14% for infected preschool-age children and 7% for the whole population) [17]. The risk of anaemia attributable to hookworm infection (4.2%) is comparable to that estimated for *S. haematobium* (3.7%) and is significantly increased in hookworm*/S. haematobium* coinfections. This is consistent with evidence suggesting that morbidity associated with these infections is more pronounced in individuals with multiple infections [21]. Hookworm and *S. haematobium* infections have the smallest attributable risks both because the relative risk for these factors is modest and more importantly because the frequency of their mean prevalence in the population is low compared to malnutrition and malaria. Nevertheless, these results suggest that hookworm and *S. haematobium* infections are also important in the aetiology of anaemia in preschool children in West Africa, and deworming should be included in programmes aimed at controlling anaemia in this age group.

We calculated that a total of 6.7 million children aged 1–4 y in Burkina Faso, Ghana, and Mali are anaemic. Our regional- and country-level estimates of number of children with anaemia are in line with estimates recently put forward by the World Health Organization in the three study countries [1]. In that regard, our study generated an important cartographic resource, providing important new information about sub-national priority areas for targeting anaemia control in the region and the quantity of resources needed in those areas (Figure 6).

### Using Predictive Parasite Infection Maps to Model Anaemia

Important uncertainties should be noted from the anaemia DHS datasets and the prediction surfaces for parasite infection used in our models, which are likely to be propagated through the modelling framework. The outcome input data from the DHS surveys (anaemia and Hb) were collected in different years (2003 for Burkina Faso and Ghana and 2006 for Mali), and the covariate input prediction surfaces for parasite infection (malaria and helminth predictive surfaces) were for 2007. In order to assess relationships between anaemia indicators and potential contributors, we assumed that there was no contraction in anaemia cases in the three countries between the year anaemia data was collected and 2007. Although this temporal disparity may not be so problematic in the case of the DHS data for Mali, it may be problematic for Burkina Faso and Ghana; an overestimation of effects in those countries could be observed particularly in areas where the effects of intervention efforts to control anaemia were substantial. However, the degree to which the observed relationships are obscured by past spatially variable intervention efforts is not quantified in the literature.

A rigorous assessment of the uncertainty associated with the mapped outputs of the input African malaria map was undertaken by [36]. This assessment provides great confidence about the input surface for the countries in our study in that the probability of correct endemicity class prediction was highest in West Africa. In this region, uncertainty was most important in small areas in southwest Ghana and northwest Mali. These latter estimates adjust for population density (using the population-weighted index in uncertainty) and reflect the co-occurrence of both low density of *Pf*PR_2–10_ surveys and large populations in these regions. Despite the fact that point predictions generated by the malaria model are reasonably accurate, the model was shown to underestimate the probability of *Pf*PR_2–10_ taking low values. This means that in low endemicity areas the *Pf*PR_2–10_ may be overestimated [36]. However, our study is located in countries where malaria endemicity is high, and therefore we do not expect this suboptimal performance to significantly affect the point values of malaria endemicity used in our models. Similarly, the results of uncertainty assessment for the helminth infection covariate surfaces give us great confidence about their use in our models. The predictive ability of endemicity class membership (<50% for *S. haematobium* infection, 10% for hookworm infection, and 5% for *S. haematobium*/hookworm coinfection) was moderately good, with all AUC values above 0.7 [37].

Finally, by using existing continental-level and other mapped layers as proxies of parasite infection, we have adopted an ecological approach to modelling anaemia prevalence and Hb. This approach was chosen because comparable individual-level infection data were not available for the study area. Instead, the mean prevalence of parasite infection was used as a proxy for the true infection status of preschool children included in the analysis. This approach provides a somewhat imprecise measurement of exposure to *P. falciparum* and helminth infection and therefore may result in regression dilution bias arising from imprecise exposure measurement, which is most likely to lead to underestimation of the observed effects of parasite infections [48]. Although the observed relationships are biologically plausible, in the absence of individually collected data it is not possible to know to what extent the magnitude of relationships represent an artefact introduced by ecological fallacy.

### Using Population Attributable Fractions to Determine the Role of Competing Factors in Anaemia

We used PAFs to represent the fraction of the total anaemia risk in the population that would not have occurred if the effect associated with the contributor of interest were absent while distributions of other contributors in the population remained unchanged [48],[49]. The PAF estimates attributable outcome and not necessarily preventable outcome numbers, as it may not be possible to remove the risk factor from the population altogether. Hence the numbers may overestimate achievable impact and are therefore measures of potential impact. An alternative statistic could have been used, namely, the population impact of eliminating a risk factor (the potential number of disease events prevented in a population over the next *t* years by eliminating a risk factor) [50].

PAF estimation is of public health significance when the risk factors being investigated are clearly the most proximal in the causal pathway and when there is consensus that the exposure is amenable to intervention [38],[51]. The nutritional factors and infections included in our anaemia model are well known to be causally related to anaemia, but as outlined above, these do not represent the complete multifactorial nature of anaemia. Haemoglobinopathies and thalassemias are importance inherited haematological conditions, particularly in the population of West Africa [52], but predictive surfaces for the sickle cell trait have only recently become available [53]. This study adopted an ecological approach to anaemia modelling in that the true infectious status of children is assigned by spatially overlaying available mapped parasite endemicity surfaces. In doing so, the estimated relative risks for these factors are prone to regression dilution bias, which may contribute to more conservative PAF estimates. In the absence of comparable individual-level data, the practical and logical limitations of including surrogate factors in PAF estimation are not trivial to assess, but our results are consistent to the only study available using individual-level data [17].

Another issue related to the interpretation and public health relevance of a PAF concerns specification of the exposure group [51]. For PAF estimation we have retained the continuous nature of the parasite surfaces to enable spatial prediction across all the areas and to avoid arbitrary categorisation of parasite endemicity surfaces, which could yield reference levels with few or no observations, resulting in PAF estimates with low power. We calculated the PAF for the mean of each parasite surface in the region, which corresponds to the fraction of total anaemia risk in the population that would have been reduced had the children been living in areas where the mean prevalence of the risk factors was very close to zero. Full consideration of continuous covariates is theoretically possible and is a matter of statistical modelling, and PAF estimates (model-based) have been developed for continuous exposures [54]. Our PAF estimation may be extended in future work to estimate a more general measure than PAF, namely, the generalised impact fraction (the fraction reduction of anaemia risk that would result from changing the current distribution of the contributing factors to some modifiable distribution) [55]. However, to set the level of reduction of the risk factor would require evidence of the effectiveness of malnutrition and parasite interventions, which is not objectively available.

Accuracy of the PAF estimates also depends on the representativeness of the input data from the population of interest and the completeness of the multivariable model. The DHS anaemia data are to the best of our knowledge the most complete and representative anaemia data available in the public domain. The anaemia data were collected using standardised methods and quality control protocols (see http://www.measuredhs.com/start.cfm). The input data used to produce smooth maps of malaria included 3,384 geo-positioned records where parasite rates had been diagnosed either using microscopy (2,764 [81.7%]) or rapid diagnostic tests (*n = *587 [17.3%]) [36]. The schistosomiasis and hookworm data were obtained in nationally representative surveys using Kato Katz and urine filtration methods [41],[42]. In PAF estimation the multivariable model needs to be as complete as possible; if one or several factors act as true confounders of the association between exposure and disease, then the crude PAF estimates are in general biased and there is a need for adjustment when estimating the PAFs [55]. Regression models allow one to take into account adjustment factors as well as interaction of exposure with some or all adjustment factors [54]. We are confident in our statistical control of confounding by adjusting our analysis for age, sex, and socio-economic factors; we also considered interactions between proximal parasite infections. However, even if one uses adjusted estimates of the relative risk, PAF estimates can be biased in the presence of unaccounted confounding factors, and overestimation of PAFs can occur [49],[56]. Malaria endemicity values may be confounded by the presence of bed net usage, which in turn is known to be influenced by socio-economic status. We found collinearity between bed net usage and socio-economic indicators in the DHS data, which provided statistical support for the inclusion of socio-economic indicators only. Furthermore, these indicators are also related to a broader group of distal factors contributing indirectly to anaemia (e.g., water, sanitation, and deworming).

The order of a variable in the causal pathway and the way it is entered in a multivariable model influence its PAF estimation [57]. The impact of different combinations of proximal infection contributors on the observed relationships with anaemia indicators was assessed by building different models (Tables 2 and 4). In so doing, we noticed the effect of variable order on the resulting coefficients, and PAF estimation was conducted based on the model with best statistical support for model complexity and fit to the data. Furthermore, indirect effects can be noticed when more distal factors impact proximal risk factors by increasing their rate or prevalence. Some of the anthropometric failures used in our models as proxies of malnutrition, and stunting, in particular, can be the result of an indirect effect of both parasite infections and malnutrition, but collinearity between these factors was not identified at variable screening.

Finally, the PAFs refer not to the general population but rather to the study population in West Africa. The results generated from an adjusted PAF model for a specific population may not fit settings in other populations [49]. PAFs in other populations may differ because of varying prevalence of risk factors and the impact of additional socio-demographic factors that were not included in the original sample [56].

### Accuracy of Geostatistical Anaemia Modelling and Potential Refinements

The frequency distributions for the predicted anaemia and Hb surfaces cover substantially smaller ranges of values than those of the DHS input data. The resulting anaemia and Hb predictive surfaces are certainly smoother than the raw data from which they are predicted because the MBG modelling approach makes predictions at unsampled locations using linear associations between covariates and the DHS survey data. This smoothing effect (or interpolation) has important repercussions on the models' ability to accurately predict anaemia endemicity over very short distances.

The models performed satisfactorily when predicting point values and endemicity classes of anaemia indicators. However, certain aspects of the uncertainty statistics are suboptimal in that the anaemia risk model tends to overestimate prevalence by 5% and the Hb model tends to underestimate Hb by 10 g/l. Nevertheless, despite the different sources of uncertainty that are embedded in the MBG modelling approach, the resulting predictive maps represent an important evidence base for operational managers of anaemia control in the region.

The computational demands of the MBG modelling approach restricted the range of modelling procedures we could utilise to improve the predictive ability of the anaemia and Hb models. A number of potential improvements to the geostatistical approach could be employed in the following ways. First, future iterations of these maps should consider the incorporation of other covariates, particularly the assessment of the additional influence of inherited blood disorders (haemoglobinopathies and thalassemias) once these become available. Second, our approach could be updated once the existing mapped surfaces have been revised with the inclusion of diagnostic uncertainty into their modelling frameworks. This is particularly important for schistosomiasis in low transmission settings [58]. Third, prediction surface uncertainty around the predicted mean of infection covariates could be incorporated in the modelling framework by modelling the distribution of probable values using a beta distribution parameterized by the predicted posterior mean and the posterior standard deviation for each parasitological survey location. Fourth, the inclusion of spatial variation of spatial dependency in anaemia risk (non-stationarity) could be another possible refinement but was considered computationally infeasible. Future iterations of the present models could incorporate non-stationarity: models could assume separate regional fixed coefficients and include a series of random coefficient models incorporating different correlation structures. Fifth, the 5×5 km resolution may not have been sufficiently precise to classify exposures, and a reduced resolution could have been chosen at the expense of computational run time. For example, an urban-rural map of 5×5 km resolution may not be sufficiently precise to define clusters as rural or urban, since settlements may vary in size across the study area. Finally, infections considered here are known to cause multiple competing morbidities, and the methods presented here could be extended to investigate spatial heterogeneity of co-morbidities attributable to malaria and helminth infections. This would involve applying a multinomial analogue of the present model. Although analysis of the spatial variation in other childhood morbidity indicators, such as stunting, fever, pneumonia, and diarrhoea, has been attempted at the national scale in Malawi [59],[60] and Burkina Faso [61], and at the continental scale in the case of paediatric fevers associated to malaria infection [62], none of these studies have investigated the differential role of malnutrition and parasite infection metrics in prevalence of co-morbidities at a regional or continental scale.

### Conclusions

The combination of anaemia and mean Hb predictive maps has allowed the identification of communities in West Africa where preschool-age children are at increased risk of morbidity. The use of anaemia maps as an alternative to aggregated country-level estimates has important practical implications for targeted control in the region and could contribute to the efficient allocation of nutrient supplementation programmes and delivery of fortified foods as well as the planning and evaluation of resource needs for geographical delivery of transfusion services for severe anaemia cases. This study shows that existing continental-level disease and other mapped layers can be used to predict anaemia risk. The development of maps indicating the geographical risk profile of anaemia controlling for malnutrition and major infections would allow assessment of the risk of anaemia due to different causes, which would in turn constitute an important evidence base to work out the best balance between interventions. In the future, these maps could be updated in subsequent methodological iterations to incorporate further modelling refinements.

## Supporting Information

Text S1 **Supplementary technical information tables.**
(0.11 MB DOC)

## Abbreviations

AUC: area under curve
CIAF: composite index of anthropometric failure
DHS: Demographic and Health Surveys
DIC: deviance information criteria
Hb: haemoglobin concentration
MBG: model-based geostatistics
PAF: population attributable fraction
*Pf* PR_2–10_: *Plasmodium falciparum* parasite rate in the 2- to 10-y age group
SD: standard deviation
SSA: sub-Saharan Africa
