# Supplementary material for: Mapping the Risk of Anaemia in Preschool-Age Children: The Contribution of Malnutrition, Malaria, and Helminth Infections in West Africa
Source: PLoS Med. 2011 Jun 7;8(6):e1000438. doi: 10.1371/journal.pmed.1000438 (PMC3110251; doi:10.1371/journal.pmed.1000438)
Supplement: Text S1 — Supplementary technical information tables. (0.11 MB DOC) [file pmed.1000438.s001.doc]

**Supplementary technical information**

*Age-standardisation of hookworm and S. haematobium prediction maps*

Prior to modelling the risk of anaemia in pre-school children (1-4 years of age) across the study region, we developed model-based geostatistical models using recent and extensive data from school-based parasitological surveys conducted in the region. These surveys were carried out in school aged children (5- 19 years of age) only. Based on these data, we performed spatial predictions for hookworm and *S. haematobium* infections to the 5-9 age group (i.e. the lowest age group). Because our study aimed at anaemia in pre-school children and prevalence for the 1-4 age group is known to be uniformly lower than that of the 5-9 age group[1] we conducted structure literature searches to inform the age-standardization of the 5-9 age group helminth infection prediction maps to the 1-4 age group. We included published studies which had reported age-prevalence profiles of hookworm and *S. haematobium* for pre-school and school age children. Table S1 summarises the studies from which data was extracted to conduct the age standardization.

| Age-prevalence profile in infants and school age children | Country | Year | Age groups included | Source |
| --- | --- | --- | --- | --- |
| Hookworm |  |  |  |  |
|  | Ghana | 1986 | 0-5 yo | [2] |
|  | Kenya | 1999 | 0-5 yo | [3] |
|  | Kenya | 1992 | 0-65yo | [4] |
|  | Zambia | 2010 | 0-6yo | [5] |
|  | India | 1969 | 0-11yo | [6] |
| *S. haematobium* |  |  |  |  |
|  | Nigeria | 2005 | 0-5yo | [7] |
|  | Nigeria | 2003 | 0-5yo | [8] |
|  | Nigeria | 2010 | 1-6yo | [9] |
|  | Tanzania | 1986 | 0-25yo | [10] |
|  | Kenya | 1988 | 0-60yo | [11] |

Age standardisation of the predicted prevalence in the 5-9 age group ($P_{5-9}$) to the 1-4 age group ($P_{1-4}$) was based on the following relationship:

$P_{1-4}=P_{5-9}*s_{i}$, where $s_{i}$ is a standardisation coefficient for parasite *i*. Based on the sources identified in Table S1, $s_{i}$took the value of 0.63 for hookworm and 0.58 for *S. haematobium*.

*General considerations*

Fixed-effects logistic regression models of anaemia and linear regression models of Hb were developed in a frequentist statistical software package (Stata version 10.1, Stata Corporation, College Station, TX). In the preliminary multivariable models, population density was not found to be significantly associated with anaemia risk nor with mean haemoglobin concentration (Hb); this variable was excluded from further analysis in the respective models (Wald’s *P*>0.2). The significant individual-level variables and the helminth infection variables were entered in the final spatial models.

All spatial models had the individual covariates plus a geostatistical random effect. Model 1 just had these elements; Model 2 also had prevalence of *S. haematobium*; Model 3 had prevalence of hookworm; Model 4 had prevalence of *S. haematobium* and prevalence of hookworm; Model 5 had prevalence of *S. haematobium* and prevalence of hookworm plus a product term for prevalence of *S. haematobium* and hookworm; and Model 6 had prevalence of *S. haematobium* and hookworm co-infection.

The outputs of Bayesian models, including parameter estimates and spatial prediction at unsampled locations, are distributions termed “posterior distributions”. The posterior distributions fully represent uncertainties associated with the parameter estimates. We summarised the posterior distributions in terms of the posterior mean and 95% Bayesian credible interval (CrI), within which the true value occurs with a probability of 95%. The variables age in months, number of members in household and infection prevalences were standardised by subtracting the mean and dividing by the standard deviation. The resulting regression coefficients for these variables represent the change in terms of standard deviations in prevalence of anaemia or mean Hb that result from a change of one standard deviation in these variables.

*Model specifications*

For the purpose of Bayesian geostatistical modelling the child anaemia status is considered a binary outcome variable *Y_i_* which was labelled *Y_i_* =1 for anaemic child and 0 for non-anaemic child. The models used assume a conditional Bernoulli model for the binary outcome variable where the probability of a child *i* being anaemic, given the location *j* of the child is given by:

*i*

*p*

*k*

*j*

*i*

*k*

*j*

*i*

*u*

*x*

*p*

*log*

+

×

+

=

∑

=

1

,

,

)

(

*β*

*α*

where *Y_i,j_* is the anaemia status of an child in location *j*, *p_i,j_* is the probability of an child being anaemic in location j, *α* is the intercept, *x_i,j_* is a matrix of covariates, *β* is a matrix of coefficients and *u_i_* is a geostatistical random effect defined by an isotropic powered exponential spatial correlation function:

,

where *d_ab_* are the distances between pairs of points *a* and *b*, and is the rate of decline of spatial correlation per unit of distance. Non-informative priors were used for *α* (uniform prior with bounds - and ) and the coefficients (normal prior with mean = 0 and precision = 1 × 10^-4^). The precision of *u_i_* was given a non-informative gamma distribution.

Similarly, for the purpose of Bayesian geostatistical modelling the child Hb concentration, we assumed that Hb concentration in the population followed a normal distribution where the Hb concentration of a child *i*, given the location *j* of the child is given by:

$$Y_{i,j}\sim Norm(m_{i,j},s_{i,j})$$

$$m_{i,j}=\alpha+\sum_{k=1}^{p} \beta_{k}\times x_{i,j}+u_{i}$$

where *Y_i,j_* is the Hb concentration of a child in location *j*, *m_i,j_* is the mean Hb concentration in location *j*, *α* is the intercept, *x_i,j_* is a matrix of covariates, *β* is a matrix of coefficients and *u_i_* is a geostatistical random effect defined by an isotropic powered exponential spatial correlation function:

,

In all models, a burn-in of 5,000 iterations was allowed, followed by 10,000 iterations where values for the intercept, coefficients and predicted probability of infection at the prediction locations were stored. Diagnostic tests for convergence of the stored variables were undertaken, including visual examination of history and density plots; convergence was successfully achieved after 5,000 iterations.

The predictions of the prevalence of anaemia and mean Hb concentration were made at the nodes of a 0.1 X 0.1 decimal degree grid (approximately 11 km^2^) by interpolating the geostatistical random effect and adding it to the sum of the products of the coefficients for the fixed effects and the values of the fixed effects at each prediction location. Values of predicted prevalence of anaemia and mean Hb at unsampled locations were stored for all male children of 35 months of age (the mean age of children included in the analysis) living in household containing 8 members (the mean household size of children included in the analysis) in rural residences. The interpolation of the random effect was done using the *spatial.unipred* kriging function in WinBUGS; the *spatial.unipred* command implements Bayesian kriging [12]where the values of predicted prevalence at unsampled locations are estimated (interpolated) independently of neighbouring values, as opposed to joint prediction which is conditional on the values of neighbouring unsampled locations. Joint prediction was not considered feasible in this study due to being extremely intensive computationally.

*Estimation of the population attributable fraction of anaemia due to helminth infections*

We estimated the *PAF* of anaemia due to a specific helminth infection using the standard formula[13]:

$PAF_{1}=\frac{P_{1}(OR_{1}-1)}{P_{1}(OR_{1}-1)+1}$ ,

where$P_{1}$ is the mean prevalence of one parasite in the 1-4 age group, and $OR_{1}$ is the prevalence-specific odds ratio (OR). The OR for the prevalence of infection with one parasite was estimated by exponentiating the mean posterior estimate of the coefficient (obtained from the spatial prediction model). In the case of models containing a product term between two parasite prevalence (Model 6), the OR for the prevalence of infection with one parasite was estimated using the following standard formula [13]:

$OR_{1}=\exp\left(\beta_{1}+\gamma_{1,2}*P_{2}\right)$,

where$\beta_{1}$ is the coefficient of the fixed effect for the prevalence of infection with one parasite, $\gamma_{1,2}$is the coefficient of the product term between the two parasite prevalences and $P_{2}$ is the mean prevalence of the other parasite. From this, we calculated the number of anaemia cases that were due to helminth infections in the study area.

*References*

1. Hotez PJ, Bundy DAP, Beegle K, Brooker S, Drake L, et al. (2006) Helminth Infections: Soil–Transmitted Helminth Infections and Schistosomiasis. Disease Control Priorities in Developing Countries 2nd ed. New York: Oxford University Press. pp. 467-482.

2. Annan A, Crompton DW, Walters DE, Arnold SE (1986) An investigation of the prevalence of intestinal parasites in pre-school children in Ghana. Parasitology 92 ( Pt 1): 209-217.

3. Brooker S, Peshu N, Warn PA, Mosobo M, Guyatt HL, et al. (1999) The epidemiology of hookworm infection and its contribution to anaemia among pre-school children on the Kenyan coast. Trans R Soc Trop Med Hyg 93: 240-246.

4. Ashford RW, Craig PS, Oppenheimer SJ (1992) Polyparasitism on the Kenya coast. 1. Prevalence, and association between parasitic infections. Ann Trop Med Parasitol 86: 671-679.

5. Siwila J, Phiri IG, Enemark HL, Nchito M, Olsen A (2010) Intestinal helminths and protozoa in children in pre-schools in Kafue district, Zambia. Trans R Soc Trop Med Hyg 104: 122-128.

6. Nawalinski T, Schad GA, Chowdhury AB (1978) Population biology of hookworms in children in rural West Bengal. I. General parasitological observations. Am J Trop Med Hyg 27: 1152-1161.

7. Opara KN, Udoidung NI, Ukpong IG (2007) Genitourinary schistosomiasis among pre-primary schoolchildren in a rural community within the Cross River Basin, Nigeria. J Helminthol 81: 393-397.

8. Mafiana CF, Ekpo UF, Ojo DA (2003) Urinary schistosomiasis in preschool children in settlements around Oyan Reservoir in Ogun State, Nigeria: implications for control. Trop Med Int Health 8: 78-82.

9. Ekpo UF, Laja-Deile A, Oluwole AS, Sam-Wobo SO, Mafiana CF (2010) Urinary schistosomiasis among preschool children in a rural community near Abeokuta, Nigeria. Parasit Vectors 3: 58.

10. Lwambo NJ, Savioli L, Kisumku UM, Alawi KS, Bundy DA (1997) The relationship between prevalence of Schistosoma haematobium infection and different morbidity indicators during the course of a control programme on Pemba Island. Trans R Soc Trop Med Hyg 91: 643-646.

11. King CH, Keating CE, Muruka JF, Ouma JH, Houser H, et al. (1988) Urinary tract morbidity in schistosomiasis haematobia: associations with age and intensity of infection in an endemic area of Coast Province, Kenya. Am J Trop Med Hyg 39: 361-368.

12. Thomas A, Best N, Lunn D, Arnold R, Spiegelhalter D (2004) GeoBUGS User Manual. Cambridge: Medical Research Council Biostatistics Unit.

13. Rothman KJ, Greenland S, Lash TL (2008) Modern Epidemiology: Lippincott, Williams, & Wilkins.
